# On Exploring Hidden Structures Behind Cervical Cancer Incidence

**DOI:** 10.1177/1073274818801604

**Published:** 2018-09-25

**Authors:** Niko Lietzén, Janne Pitkäniemi, Sirpa Heinävaara, Pauliina Ilmonen

**Affiliations:** 1 Department of Mathematics and Systems Analysis, Aalto University School of Science, Espoo, Finland; 2 Institute for Statistical and Epidemiological Cancer Research, Finnish Cancer Registry, Helsinki, Finland; 3 Department of Public Health, University of Helsinki, Helsinki, Finland

**Keywords:** cancer incidence, cervical cancer, invariant coordinate selection, time series analysis, menopause

## Abstract

Finding new etiological components is of great interest in disease epidemiology. We consider time series version of invariant coordinate selection (tICS) as an exploratory tool in the search of hidden structures in the analysis of population-based registry data. Increasing cancer burden inspired us to consider a case study of age-stratified cervical cancer incidence in Finland between the years 1953 and 2014. The latent components, which we uncover using tICS, show that the etiology of cervical cancer is age dependent. This is in line with recent findings related to the epidemiology of cervical cancer. Furthermore, we are able to explain most of the variation of cervical cancer incidence in different age groups by using only two latent tICS components. The second tICS component, in particular, is interesting since it separates the age groups into three distinct clusters. The factor that separates the three clusters is the median age of menopause occurrence.

## Introduction

Increasing cancer burden has made researchers worldwide search for factors that explain the increasing trends.^1^ In addition to age, period, and cohort, several other observable factors have an effect on the trends in cancer incidence. Improved diagnostics, cancer screening programs, and general awareness have increased the incidence of many cancers. Additionally, several lifestyle-related factors have an effect on the cancer incidence. For example, changes in smoking prevalence have a clear delayed effect on the incidence of lung cancer. However, there are also unknown and unobservable underlying factors that have effects on cancer incidence rates. Identification and quantification of these unknown factors would further help in understanding the trends in cancer incidence data.

In this article, we consider a time series version of invariant coordinate selection (tICS) in the context of latent components of calendar time variation in incidence. Invariant coordinate selection is closely related to the more famous independent component analysis (ICA). Under certain assumptions, the ICS procedure provides a solution to the independent component problem. The objective in ICS is to transform the observed data into an invariant coordinate system. Occasionally, the new coordinate system reveals structures from the data that are not visible in the original coordinate system. The clear advantage of ICS, when compared to, for example, the frequently used principal component analysis (PCA), is that the chosen scales and units of measurement have no effect on the results.

Invariant coordinate selection was presented as an exploratory tool for searching interesting features from independent and identically distributed (i.i.d.) multivariate data.^2,3^ In this article, we present a time series version of ICS, denoted by tICS. Similarly as in the i.i.d. case, under certain assumptions, the method provides a solution to the time series ICA (or blind source separation [BSS]) unmixing problem. Independent component analysis has been applied successfully to analyze, for example, electroencephalography (EEG) data sets of the brain^4^ and to cluster mammogram data sets.^5^ In this article, we however do not assume an underlying ICA model. Instead of that, we, in the spirit of Tyler et al,^3^ apply tICS as an exploratory tool in the search of hidden underlying structures from cancer incidence data.

We apply the tICS transformation to a cervical cancer incidence data set. The data set is from Finland between the years 1953 and 2014 and it is available online.^6,7^ Incidence of many cancers has increased in Finland. However, an effective cervical cancer screening program has enabled to treat precancerous conditions, and because of that, the cervical cancer incidence rate has decreased.^8,9^

### Ethical Considerations

The data set used in this study is available in the public domain and can be accessed from the web site of the NORDCAN database.^6,7^ Furthermore, the corresponding data set is provided as Supplemental Material.

## Invariant Coordinate Selection

In this section, we review the scatter matrix-based ICS method introduced in the study by Tyler et al.^3^

Let *X* ∈ **R** *^n×p^*, where, *n > p*. A location statistic $\hat{T}$ *(X)* is a *p*-vector valued statistic, that is affine equivariant in the sense that

$$\hat{T}\left(XA+1_{n}b^{T}\right)=A^{\text{T}}\hat{T}\left(X\right)+b,$$

for all nonsingular *p × p* matrices *A* and for all *p*-vectors *b*. A scatter matrix $\hat{S}\left(X\right)$ is a positive definite *p × p* matrix valued statistic, that is affine equivariant in the sense that

$$\hat{S}\left(XA+1_{n}b^{T}\right)=A^{\text{T}}\hat{S}\left(X\right)A,$$

for all nonsingular *p × p* matrices *A* and for all *p*-vectors *b*.

Elementary examples of a location statistic and a scatter matrix are the sample mean vector and the sample covariance matrix. There are several other location statistics and scatter matrices, even families of them, that have different desirable properties, for example, robustness, efficiency, limiting multivariate normality, and computational efficiency.^10-12^

Let $\hat{T}$_1_ *(X)* denote an arbitrary but fixed location statistic, and let ${\hat{S}}_{1}\left(X\right)$ and ${\hat{S}}_{2}\left(X\right)$ denote arbitrary but fixed and different scatter matrices. The ICS transformation $\hat{\text{Γ}}\left(X\right)$ for the data *X* is defined such that if

$$Z^{\text{T}}=\hat{\text{Γ}}\left(X\right)\left(X^{\text{T}}-{\hat{T}}_{1}\left(X\right)1_{n}^{\text{T}}\right),$$

then

$${\hat{T}}_{1}\left(Z\right)=\;0,\;{\hat{S}}_{1}\left(Z\right)=I_{p},\;{\hat{S}}_{2}\left(Z\right)=L=\text{diag}\left(l_{1},\;\ldots ,l_{p}\right),$$

where $\left|l_{1}\right|\geq \left|l_{2}\right|\geq \ldots \geq \left|l_{p}\right|.$

If the data is continuous, then the transformation matrix $\hat{\text{Γ}}\left(X\right)$ is almost surely unique up to the signs of its row vectors. Consequently, it is affine equivariant up to the signs, and it can be used to transform the data to up to sign invariant coordinates. Thus, affine transformations to the original data have no effect on the procedure. The transformation ensures that when examining the transformed data, the findings are true findings and not artefacts of the chosen coordinate system. Note that whereas PCA makes data uncorrelated, ICS makes data independent with respect to two measures of dependence. Invariant coordinate selection transformation can be seen as affine invariant PCA that, on top of first-order dependencies, considers second-order dependencies as well. In other words, PCA transforms the data into a coordinate system, where the coordinates are diagonal with respect to the covariance matrix. On the other hand, ICS transforms the data into a coordinate system, where the corresponding two scatter matrices are diagonal. Moreover, whereas PCA is highly affected by scaling of the variables, ICS, due to affine invariance, is not affected by scaling at all.

It can be shown that if the chosen location and scatter estimates converge, so do the statistics $\hat{\text{Γ}}$ and *L*. Moreover, under the assumption of asymptotic normality of the location and scatter estimates, the statistics $\hat{\text{Γ}}$ and *L* are also asymptotically normal.^13-15^

The scatter matrix based ICS transformation was first introduced in the context of ICA.^16^ It was based on the use of the regular covariance matrix and the scatter matrix based on fourth moments. The transformation was named the fourth-order blind identification transformation. Later, the ICS transformation was considered in wider settings.^3^ In the independent component model, the elements of a random *p*-vector are assumed to be linear combinations of the elements of an unobservable *p*-vector with mutually independent components. The aim in ICA is to recover the independent components by estimating an unmixing matrix that transforms the observed *p*-vector to independent components.^17^ If a scatter matrix is diagonal for all random vectors with independent components, then we say that the corresponding scatter matrix has the independence property. Assuming that the chosen scatter matrices have the independence property, the ICS procedure provides a solution for the ICA problem.^14,16,18,19^ Under the assumption of i.i.d. observations, the use of the scatter matrix-based ICS transformation has not been limited to ICA. It has been applied, for example, in finding hidden underlying structures of data, in constructing affine invariant depth functions, in dimension reduction, in analyzing mixture models, and in defining multivariate skewness and kurtosis measures.^3,13-15,20^

For time series data, we can obtain transformations similar to ICS, by replacing the second scatter matrix in the transformation by an autocovariance matrix. Depending on the data set, we could also use two autocovariance matrices with different lags. In the context of second-order stationary time series, the procedure is called the algorithm for multiple unknown signals extraction (AMUSE).^21^ Like the scatter matrix-based ICA was extended to ICS, we consider applying AMUSE transformation in wider settings. We use it in dimension reduction and as an exploratory tool in the search of hidden structures in our case study of cancer incidence data.

## Invariant Coordinate Selection for Time Series Data

In this section, we examine autocovariance matrix-based transformations that have previously been considered in the case of second-order stationary time series.^21,22^

Let *X* ∈ **R** *^n×p^*, *n > p*, be an observed *p*-variate time series where the components of *X* are continuous stochastic processes. The time series *X* contains *n* ordered observations and we denote the *i*th observation by *x_i_*, such that $x_{i}\in R^{p}$ for every i∈{1,2,…, n}. Likewise, we denote the *k*th component, that is, *k*th column of *X*, by x(k), where $x\left(k\right)\in R^{n}$ for every k∈{1,2,…,p}. Note that the word component here (as in PCA) refers to the new coordinates given by the tICS transformation. Let τ∈{0,1,…,n−1} and let $\hat{T}\left(X\right),\;{\hat{S}}_{0}\left(X\right)$, and ${\hat{S}}_{\tau }\left(X\right)$ denote the sample mean vector, the sample covariance matrix, and the sample autocovariance matrix with lag τ, respectively, that are computed from *X*. The sample autocovariance matrix is defined as

$${\hat{S}}_{\text{τ}}\left(X\right)=\;\frac{1}{\left(n-\text{τ}\right)}\;\overset{n-\text{τ}}{\underset{j=1}{\sum }}(x_{j}-\hat{T}\left(X\right)){\left(x_{j+\text{τ}}-\hat{T}\left(X\right)\right)}^{\text{T}},\;$$

where the sample covariance matrix is obtained with τ = 0, up to a constant.

In our approach, we use the symmetrized version of the autocovariance matrix estimator. Note that the eigenvectors of symmetric matrices are more stable to estimate when compared to the estimation of the eigenvectors of nonsymmetric matrices. One could alternatively use nonsymmetric autocovariance matrices here. The symmetrized sample autocovariance matrix is defined as

$${\hat{S}}_{\tau }^{\text{S}}=\frac{1}{2}\left({\hat{S}}_{\tau }+{\hat{S}}_{\tau }^{\text{T}}\right),$$

where ${\hat{S}}_{\tau }^{\text{S}}$ always produces symmetric estimates.

The time series ICS transformation matrix, that is, the unmixing matrix, $\hat{\text{Γ}}\left(X\right)$ for the data *X* is now defined such that if

$$Z^{\text{T}}=\hat{\Gamma }\left(X\right)\left(X^{\text{T}}-\hat{T}\left(X\right)1_{n}^{\text{T}}\right),$$

then

$$\hat{T}\left(Z\right)=0,\;{\hat{S}}_{0}\left(Z\right)={\text{I}}_{\text{p}},\;{\hat{S}}_{\tau }^{\text{S}}\left(Z\right)=\text{Λ},$$

where $\text{Λ}=\text{diag}\left({\text{λ}}_{1},\ldots ,\lambda_{\text{p}}\right)$ and $\left|{\text{λ}}_{1}\right|\geq \ldots \geq \left|\lambda_{\text{p}}\right|>0.$

For general time series data, we call the transformation time series ICS or shortly tICS. The tICS transformation transforms time series data to invariant coordinates and it may be used in dimension reduction and/or as an exploratory tool in the search of hidden structures from time series data. We can think that ICS is an extension to PCA and tICS is incorporating the time series structure to the ICS transformation.

The feasibility of the tICS procedure depends strongly on the choice of the lag parameter τ. The approach proposed in literature is to try different values of τ and choose the lag parameter such that the estimate Λ has as distinct diagonal elements as possible.^23^

Note that,

$${\hat{\text{S}}}_{0}{\left(X\right)}^{-1}{\hat{S}}_{\tau }^{\text{S}}\left(X\right)\hat{\text{Γ}}{\left(X\right)}^{\text{T}}=\hat{\text{ Γ}}{\left(X\right)}^{\text{T}}\text{ Λ},$$

that is, the diagonal elements of Λ are the eigenvalues of ${\hat{S}}_{0}{\left(X\right)}^{-1}{\hat{S}}_{\tau }^{\text{S}}\left(X\right),$ and the column vectors of $\hat{\text{Γ}}{\left(X\right)}^{\text{T}}$ are the corresponding eigenvectors. If the diagonal elements of Λ are distinct, then the solution is unique up to the signs of the eigenvectors. If the underlying stochastic processes are continuous, then the transformation matrix $\hat{\text{Γ}}\left(X\right)$ is almost surely unique up to the signs of its row vectors. Consequently, it is affine equivariant up to the signs, and it can be used to transform time series data to invariant coordinates.

The eigenvalues of the tICS transformation can be seen as relative autocovariances (with lag τ) of the tICS components, when the variances have been standardized to be equal to 1. For further details regarding the interpretation of the eigenvalues, see section 3 of the study by Tyler et al.^3^

We refer to the columns of *Z* as the estimated tICS components. After deriving the estimate $\hat{\text{Γ}}\left(X\right)=\hat{\text{Γ}}\in R^{p\times p},$ the observed centered curves can be estimated using the inverse ${\hat{\text{Γ}}}^{-1}$ such that,

$$\begin{array}{c} {\hat{x}}_{t}\left(j\right)=\overset{q}{\underset{k=1}{\sum }}z_{t}\left(k\right){\left[{\hat{\text{Γ}}}^{-1}\right]}_{jk},t\in \left\{1,\ldots ,n\right\}\text{and}\;j,k,q\in \left\{1,\ldots ,p\right\},\left(1\right) \end{array}$$

where $z_{t}\left(k\right)$ is the *k*th estimated tICS component at time point *t*, *q* is the chosen number of tICS components used in the estimation, and ${\hat{x}}_{t}\left(j\right)$ is the resulting estimate for the observed *j*th time series at time *t*. Note that if we use all the tICS components, that is, choose *q = p*, the estimates are then exactly the centered versions of the original time series. Furthermore, the *q* components used in the estimation are the components that have the largest absolute diagonal elements on the estimated matrix Λ.

Note that, the AMUSE estimator is known to converge under the assumption of stationary BSS model.^22^ If the assumption does not hold, then it is not known whether the estimators converge or not. However, even under nonstationary data, the corresponding estimators provide transformations into invariant coordinates.

In this article, we apply the above-described method to cancer incidence data. Note that, one could examine underlying structures of cancer incidence data by applying other ICA and BSS type approaches. One does not have to limit to transformations that are based on using two autocovariance matrices. One can replace the autocovariance matrices with other time-dependent scatter matrices (eg affine equivariant spatial sign autocovariance matrices^24^). Moreover, one could also consider joint diagonalization of several (more than two) time-dependent scatter matrices, or other similar approaches, see for example.^25-27^

## Underlying Trends in Cancer Incidence

In this section, we apply time series ICS transformation to time series data of age-stratified cervical cancer incidence rates between the years 1953 and 2014 in Finland. The data set was obtained from the Finnish population-based cancer registry that has excellent data quality and coverage of registration of solid tumors.^28,29^ The data are available on the web page of the NORDCAN project.^6,7^

During this 60-year period, cervical cancer incidence has been affected mostly by the nationwide screening program.^30^ The organized cervical cancer screening program was introduced in 1963 and it reached full coverage of Finland during the decade. Municipalities are responsible for inviting females between the ages of 30 and 60 for inspection every 5 years. In some municipalities, invitations are extended also to females aged 25 and/or 65 years. Thus, we have divided the data into 5-year age groups. We search for underlying structures that can be used in describing changes in cancer incidences over the years.

**Figure 1.**
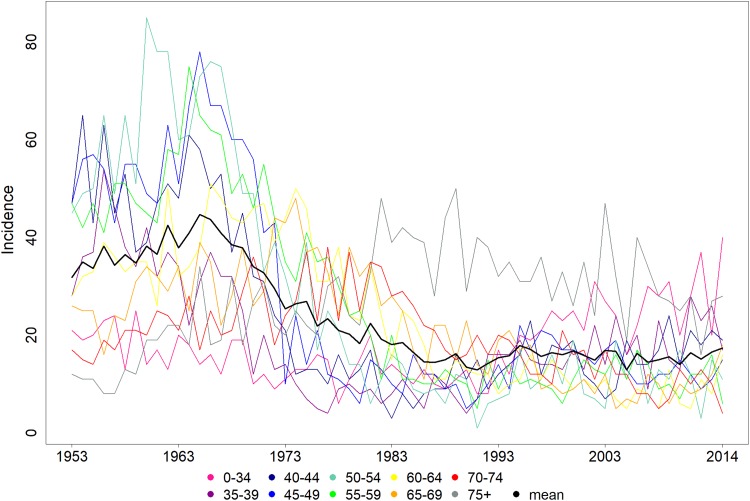
Age-stratified cervical cancer incidence in Finland between 1953 and 2014.

**Figure 2.**
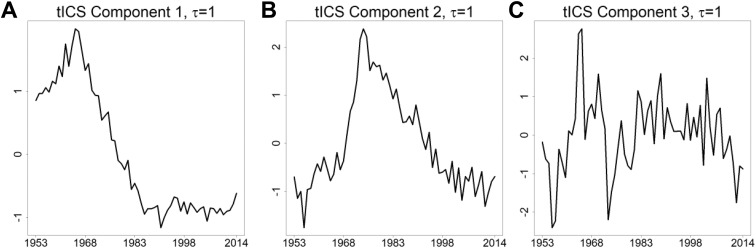
The first three estimated tICS components for 1 = 1. A, First tICS comp. B, Second tICS comp. C, Third tICS comp. tICS indicates time series version of invariant coordinate selection.

Due to the sparse number of cancer incidence in the age groups of younger than 35, they have been combined into a single group. Likewise, age groups of older than 74 have been combined into a single group. Thus, our data set contains 10 separate age groups, resulting in a 10-dimensional time series with 62 observations. The age groups with the lowest mean and median incidence are under 35, 70 to 74, and over 75. Annual incidences of the different age groups are presented in Figure 1. The incidences in Figure 1 are all positive as the figure displays the actual observed noncentered incidences. The sample mean time series of the cervical cancer incidence is presented as a black curve in Figure 1.We performed the tICS transformation using the sample covariance matrix as the first scatter matrix and the sample autocovariance matrix with lag parameter as the second scatter matrix. The first three estimated tICS components are presented in Figure 2. We want to emphasize that the scales of the tICS components are not relevant. Instead, we seek for curves that have interesting shapes. The first three components have the largest corresponding absolute diagonal values on the estimated matrix Λ, and thus, they are the most important. The remaining seven tICS components resemble noise and are presented in the Online Appendix.

The shape of the first component is similar to the mean curve time series, compare the black curve in Figure 1 and the curve in Figure 2A. We name the first component as “the average.” The first component represents the average cervical cancer incidence.

The shape of the second tICS component is the most interesting (see Figure 2B). It represents increasing trend from 1953 until mid-70s and a decreasing trend after that. We call the second component “the turning point.”

The third tICS component in Figure 2C is less interesting when compared to the first tICS components. Like the last seven components, the third component has a great deal of resemblance to random variation, with no systematic behavior.

**Figure 3.**
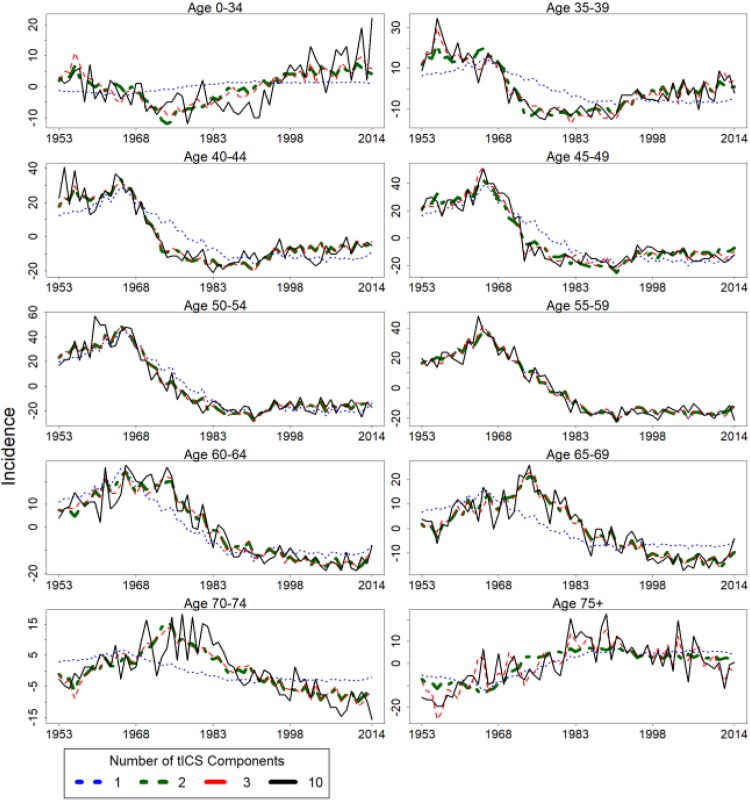
Cervical cancer incidence in Finland between 1953 and 2014 in terms of the estimated components. The estimation is performed using Equation 1. Note that the black curve, that is estimated using all of the tICS components, is the centered version of the corresponding incidence curve in Figure 1. tICS indicates time series version of invariant coordinate selection.

**Figure 4.**
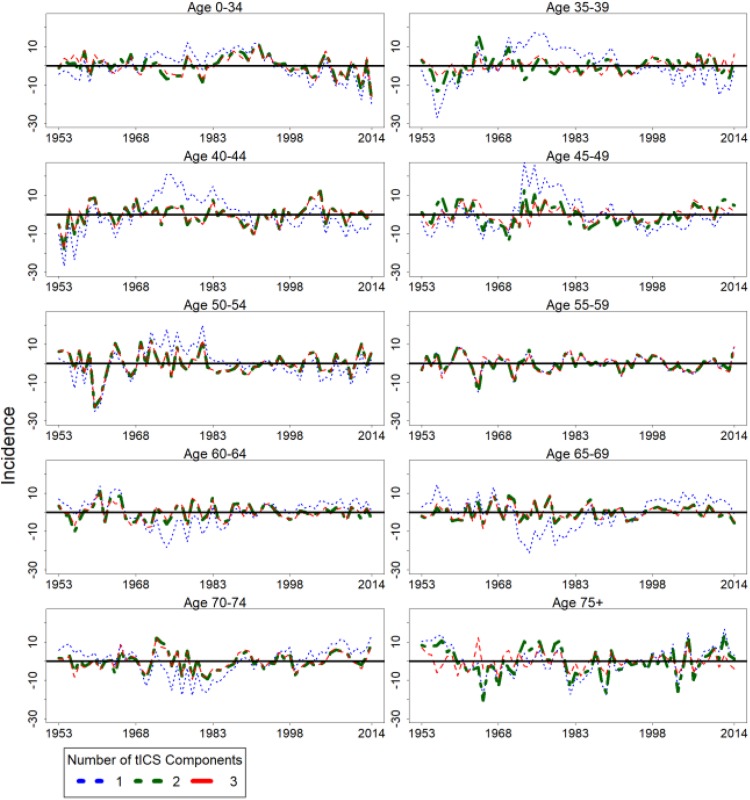
The differences between the observed cervical cancer incidence curves and the estimated incidence curves using the tICS components. The tICS components are estimated using Equation 1. tICS indicates time series version of invariant coordinate selection.

The incidence curves in different age groups can be roughly estimated using only the first two components, see Equation (1) and the green curves in Figures 3 and 4. In Figures 3 and 4, the green curves have almost identical trajectories compared to the red curves, where the red curves are the estimated incidence curves using three tICS components. This is an indication of the third tICS component not providing significant improvement in explaining the variation of the original cervical cancer incidences. The age groups of under 35, 70 to 74, and over 75 have the lowest mean incidence. Thus, random variation has a larger effect in these age groups, which could be the reason for the estimates being worse when compared to the other age groups.

**Figure 5.**
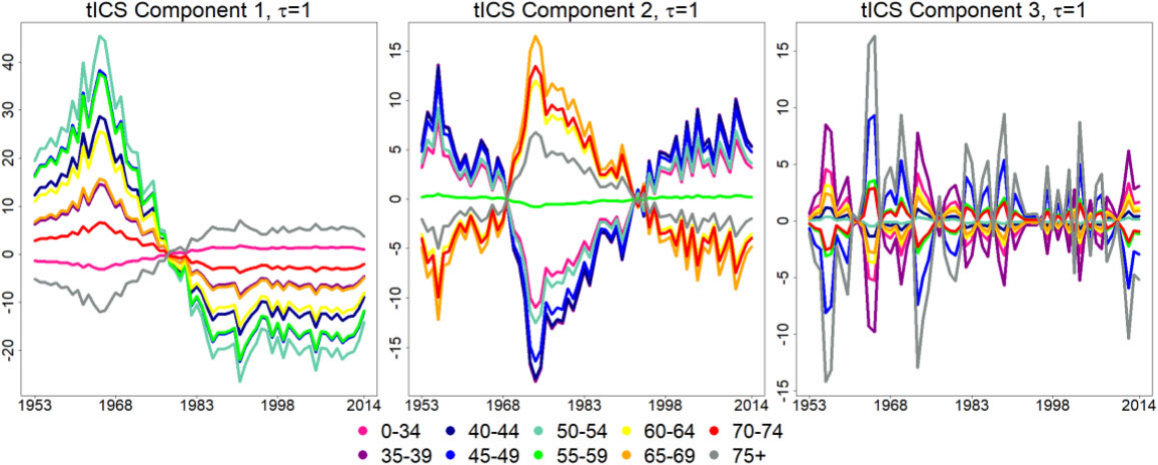
Clustered age-stratified cervical cancer incidences for the first three tICS components. The curves are the tICS components multiplied with the corresponding loadings. tICS indicates time series version of invariant coordinate selection.

In order to visually observe cluster structures, the scores of the components, that is, the curves

$$c_{\mathrm{ik}}=z_{t}\left(\text{k}\right){\left[{\hat{\text{Γ}}}^{-1}\right]}_{ik},\;k\in \left\{1,2,3\right\},\;i\in \left\{1,\ldots ,p\right\},$$

are presented in Figure 5, where *p* = 10 is the number of age groups in our case study. We refer to the value ${\left[{\hat{\text{Γ}}}^{-1}\right]}_{ik}$ as the loading related to the age-group *i* and the tICS component *k*. If the absolute value of the loading is large, that specific tICS component has a high impact in explaining the variation of the specific age-group. The curves with the highest absolute loadings are the top and bottom curves in Figure 5. Likewise, low absolute loading values indicate that the specific tICS component has a low impact in explaining the variation of the specific age-group. The curves with low absolute loadings are the middle curves in Figure 5. The curves $c_{ik}$ provide visual clustering based on the first 3 components.

We want to further emphasize that the curves inside a single image of Figure 5 are all the same up to a constant. Hereby, the negative and positive signed curves will always intersect at some point of Figure 5 (the tICS components are centered and none of the are constant). The figure is formed such that, for example, for the time series of the age-group 65 to 69 for tICS component 2, we take the second tICS component, that is presented in Figure 2B, and multiply it with the corresponding loading, that is ${\left[{\hat{\text{Γ}}}^{-1}\right]}_{82}$ here. The loading ${\left[{\hat{\text{Γ}}}^{-1}\right]}_{82}$ is the largest (positive) and the loading ${\left[{\hat{\text{Γ}}}^{-1}\right]}_{22}$, which corresponds to the age-group 35 to 39, is the smallest (negative) loading among the loadings ${\left[{\hat{\text{Γ}}}^{-1}\right]}_{\text{i}2},\text{ i }\in \left\{1,\ldots ,\;10\right\}$, which are the loadings related to the second tICS component. The largest and smallest loadings can be conveniently verified from, for example, the peak of the second tICS component in Figure 5.

The first set of curves, $c_{\cdot 1}$, are ordered based on the trend of cancer incidence in a specific age-group. The curves representing age groups, where the incidence has been decreasing, that is, the behavior is similar to the mean curve, have a positive loading in this component. The largest positive loading is for the age-group 50 to 54, which is the age-group where the incidence has decreased the most. The age groups that have a negative loading with respect to the first component have the first tICS component mirrored in Figure 5. The age-group of older than 75 has the largest negative loading with respect to the first component. The behavior of the incidence in this age-group is the opposite compared to the mean cancer incidence (see Figure 1).

The second set of curves, $c_{\cdot 2}$, provide clustering based on the second component in Figure 5. Visual clustering reveals that the second component splits the age groups according to the age of menopause. Age groups of older than 60 have a positive loading, the age-group of 55 to 59 has a loading close to zero, and the age groups younger than 50 have a negative loading.

Visual clustering based on the remaining components, including the third tICS component in Figure 5, reveal nothing interesting, as was expected from random variation.

## Discussion

In our case study of cervical cancer incidence in Finland, tICS produced interesting findings. The uncovered latent structures support recent findings discovered using other methods.^9^ The first component clustered the age groups with respect to trend. It separated the age groups where cancer incidence has been decreasing from those where the incidence has been increasing or has stayed relatively same. The information provided by the clustering of the first component could also be easily be verified from Figure 1, and thus, the clustering provided by this component is not particularly interesting. The components after the second one seemed to be random variation, that is, uninteresting noise. The tICS components 4 to 10 are presented in the Online Appendix.

The loadings with respect to the second tICS component clustered the different age groups into three separate clusters. The average age of the occurrence of menopause is the factor that separates these clusters. Finnish women usually experience menopause between the ages 45 and 55, where the median age of natural menopause has been estimated to be 51.^31^ The first cluster contains age groups with negative loadings, which are the groups of younger than 55. The second cluster contains age groups with positive loadings, which are the groups of older than 59. The third cluster contains only the age-group of 55 to 59 and this group has an almost zero loading with respect to the second component.

The behavior of the second component supports the findings of the calendar time varying contribution of early and late age-related components in cervical cancer.^9^ The first crossing point of the curves is soon after starting the cervical cancer screening in 1963. Hidden structures in the incidence in age groups close to menopause are different from those in the age groups far away from menopause. This cooccurs at same age as menopause, suggesting potential role of hormonal changes in the etiology of cervical cancer.

For future work, we could consider alternative stratifications in our analysis, for example, stratification according to cohort. Furthermore, we could try to find new explaining variables that have similar shapes as the second tICS component. We could end up finding previously ignored variables that give us new insight into the etiology of cervical cancer.

Generally, the tICS procedure is sensitive to the choice of the lag parameter τ. In order to solidify our findings, we performed the tICS procedure with several different values of τ, and the best separation was obtained using lag τ = 1. The corresponding lag produced the most distinct values for the diagonal elements of the estimated matrix Λ and the best estimated curves in Figures 3 and 4. However, the most interesting findings - the cluster structures visible in Figure 5 - stayed almost identical with lag parameters τ=1, 2, 3, 5. Hereby, in our case study, the procedure was not highly sensitive to changes in the choice of τ. The results with lag parameters τ=2, 3, 5, 15 are presented in the Online Appendix.

For comparison, we also applied the second-order blind identification (SOBI)^25^ procedure. In SOBI, the second diagonalization is replaced with a joint diagonalization with respect to multiple autocovariance matrices with distinct lags. This makes the choice of the lag parameter less decisive. The shapes of the first two components were similar to our findings. We performed a similar estimation using the first three SOBI components as in Figures 3 and 4. The performance of the first two and three SOBI components, with every set of lags that we tried, was considerably worse in explaining the variation of the centered versions of the original time series. One can always diagonalize multivariate data with respect to two scatter matrices. However, if one is outside of ICA/BSS settings, diagonalization of more than two scatter matrices is not always possible. This could explain that the SOBI method gives worse results here. Thus, we decided to use the tICS procedure instead of SOBI in this case study.

Since the tICS procedure is affine invariant, it ensures that the findings are not simply artifacts of the used coordinate system. Exploratory tools such as PCA are not affine invariant. Affine transformations of the original data would yield completely different results in PCA, whereas tICS would remain unaffected. For further comparison, we also applied the PCA transformation to this data set. The first two principal components were similar in shape to the first two tICS components. However, like in the case of SOBI, the estimation using the first two and three principal components yielded worse results in comparison to the estimation of the first two and three tICS components visible in Figures 3 and 4.

We are facing increasing cancer burden in Western countries.^1^ There have been extensive studies of the risk factors of specific cancers. However, attributable fraction to known risk factors is often quite low, leaving us with the need of further understanding the etiology. Identification of latent components of cancer incidence may open new prospects. Furthermore, if age-related latent components explaining the cancer incidence rates are modifiable, they are important in future efforts of reducing cancer burden in Finland and worldwide.

## Supplemental Material

Supplemental Material, cervical2014 - On Exploring Hidden Structures Behind Cervical Cancer Incidence Click here for additional data file. Supplemental Material, cervical2014 for On Exploring Hidden Structures Behind Cervical Cancer Incidence by Niko Lietzén, Janne Pitkäniemi, Sirpa Heinävaara, and Pauliina Ilmonen in Cancer Control

## Supplemental Material

Supplemental Material, Rcode - On Exploring Hidden Structures Behind Cervical Cancer Incidence Click here for additional data file. Supplemental Material, Rcode_(1) for On Exploring Hidden Structures Behind Cervical Cancer Incidence by Niko Lietzén, Janne Pitkäniemi, Sirpa Heinävaara, and Pauliina Ilmonen in Cancer Control
