# Supplementary material for: On Exploring Hidden Structures Behind Cervical Cancer Incidence
Source: Cancer Control. 2018 Sep 25;25(1):1073274818801604. doi: 10.1177/1073274818801604 (PMC6156216; doi:10.1177/1073274818801604)
Supplement: Supplemental Material, Rcode - On Exploring Hidden Structures Behind Cervical Cancer Incidence [file Rcode.pdf]

}

## Cervical-Fun.R

```
##########################################################################
#
# R-code that contains the necessary functions for producing the
# figures of the paper "On Exploring Hidden Structures Behind Cervical
# Cancer Incidence"
#
# Author:
# email:
##########################################################################

##########################
# AMUSE related functions
##########################

# X        -        Positive definite square matrix from which the square root is calculated from

msqrt <- function(X){ # Returns the symmetric matrix square root
  eigen(X)$vectors %*% (diag(eigen(X)$values)^(1/2) %*%Conj(t(eigen(X)$vectors)))
}

# X        -        Data set from which the regular covariance matrix is calculated from

cov1 <- function(X){ # Returns the covariance matrix
  cent <- sweep(X,2,colMeans(X),"-")
  return(1/(dim(X)[1]-1)*crossprod(cent,Conj(cent)))
}

# X        -        Data set from which the autocovariance matrix is calculated from
# tau      -        Lag parameter for the autocovariance matrix
```

```
acov <- function(X,tau){ # Returns the autocovariance matrix using lag tau
  n <- dim(X)[1]
  Z <- sweep(X,2,colMeans(X),"-")
  tmp1 <- crossprod(Z[1:(n-tau),],Conj(Z[(1+tau):n,]))
  AC <- 1/(2*(n-tau)) * (tmp1 + t(Conj(tmp1)))
  return(AC)
}

# D		-		Data set where the AMUSE procedure is applied to
# tau		-		Lag parameter for the AMUSE transformation

NAMUSE <- function(D,t){ # Returns the AMUSE transformed data and the AMUSE transformation matrix
  n <- dim(D)[1]
  p <- dim(D)[2]
  cent <- sweep(D,2,colMeans(D),"-")
  S1 <- cov1(D)
  COV.sqrt.i <- solve(msqrt(S1))
  Z <- tcrossprod(cent,COV.sqrt.i)
  S2 <- acov(Z,t)
  U2 <- eigen(S2,symmetric=TRUE)$vectors
  G<- crossprod(Conj(U2),COV.sqrt.i)
  DT <- Z %*% Conj(U2)
  L <- list(Gamma=G,Data=DT)
  return(L)
}

########################################
# Functions for generating the figures
########################################
```

```
# Function for placing the legends and axis labels to correct positions
reset <- function() {
  par(mfrow=c(1, 1), oma=rep(0, 4), mar=rep(0, 4), new=TRUE)
  plot(0:1, 0:1, type="n", xlab=" ", ylab="", axes=FALSE)
}

# D        -        Original time series
# U        -        AMUSE transformed data
# UM       -        AMUSE transformation matrix
# col      -        Vector containing the colors for the curves
# lab      -        Vector containing the labels for the titles and legends
# ncom     -        Number of tICS component plot figures generated
# tau      -        Lag parameter of the AMUSE transformation (used in the titles of the figures)
# nam      -        Name for the output figures

canplot <- function(D,U,UM,col,lab,ncom,tau,nam){ # Generates the figures to current working directory

  str=1953

  n <- dim(D)[1]
  p <- dim(D)[2]

  meancurve <- ts(as.matrix(rowMeans(D)),start=str)

  ticsvec1 <- as.integer(seq(from=1953,to=2014,length.out=7))

  postscript(paste0(nam,"original.eps"),horizontal = FALSE,paper="special",height=16,width=23)
  par(mar = c(1.5, 1.5, 2.0, 1.5), oma = c(4, 2.5, 0.4, 0.4))
  par(tcl = -0.25)
  par(mgp = c(2, 0.6, 0))
  par(mfrow=(c(1,1)),cex=2.5)
```

```
ts.plot(D,gpars=list(axes=FALSE,col=col,type="l",lwd=2,xlab=NA,ylab=NA))
lines(meancurve,col="#000000",lwd=4)
mtext("Incidence", side = 2, outer = TRUE, cex = 4, line = 1,col = "#000000")
title("Cervical Cancer Incidence in Finland between 1953-2014",cex.main=1.8,font.main=1)
axis(2,cex.axis = 1.45,lwd.ticks = 0)
axis(1, labels=ticsvec1,at=ticsvec1,cex.axis = 1.45)
box()
reset()
legend(x="bottom",legend = c(lab,NA,"mean"), col=c(col,NA,"#000000"),
    pch=16,ncol=6,bty="n", cex = 3.5)
dev.off()

ticsvec2 <- as.integer(seq(from=1953,to=2014,length.out=5))

for(i in 1:ncom){

  plotnam <- paste(nam,"comp",i,".eps",sep="")
  titnam<- bquote(paste("tICS Component ",.(i),", ",tau, "=",.(tau),sep=""))

  axmax <- as.integer(max(U[,i]))
  axmin <- as.integer(min(U[,i]))
  axseq <- seq(from=axmin,to=axmax,by=1)

  postscript(plotnam,paper="special",horizontal = FALSE,height=12,width=12)
  par(mar = c(1.5, 1.9, 2.2, 1.5), oma = c(0.25, 0.25, 0.25, 0.25))
  par(tcl = -0.25)
  par(mgp = c(2, 0.7, 0))
  par(mfrow=(c(1,1)),cex=2.5)

  ts.plot(ts(U[,i],start=str),gpars=list(axes=FALSE,type="l",lwd=6,ylab=NA))
  title(titnam,cex.main=2.5)
```

```
  axis(side=2,cex.axis = 1.75,labels=axseq,at=axseq)
  axis(side=1,labels=ticsvec2,at=ticsvec2,cex.axis = 1.75)
  box()
  dev.off()
}

xplotc <- 0.0495
yplotc <- 0.15

inv <- solve(UM)

postscript(paste0(nam,"estim.eps"),horizontal = FALSE,paper="special",height=24,width=20)
par(mfrow = c(6, 2))
par(cex = 1.5)
par(mar = c(2.5, 1.5, 1.75, 1.5), oma = c(2, 4, 0.5, 0.5))
par(tcl = -0.25)
par(mgp = c(2, 0.95, 0))

for(i in 1:p){
  cervplot = matrix(data=NA, nrow=n, ncol=4)
  tmp <- 0
  for(k in 1:3){
    tmp <- tmp + inv[i,k] * U[,k]
    cervplot[,k] = tmp
  }

  cervplot[,4] = U %*% inv[i,] # This is equal to : D[,i] - mean(D[,i])
  cervplot.ts <- ts(as.matrix(cervplot),start=str)
  titnam <- paste("Age ",lab[i],sep="")

  ts.plot(cervplot.ts,gpars=list(xlab=NA,ylab=NA,axes=FALSE,
```

```
                    col=c("#0000FF","#006400","#FF0000","#000000"),
                    lty=c(3,4,2,1),lwd=c(3,8,3,3)))

  title(titnam,cex.main=2,font.main=1)
  axis(side=2,cex.axis = 1.75)
  axis(side=1,labels=ticsvec2,at=ticsvec2,cex.axis = 1.75)
  box()

}
mtext("Incidence", side = 2, outer = TRUE, cex = 4, line = 2.2,col = "#000000")
reset()

legend(x=xplotc,y=yplotc, legend = c(1:3,10),
    col=c("#0000FF","#006400","#FF0000","#000000"),lwd=12,
    lty=c(3,4,2,1),horiz = TRUE,
    cex = 3,title="Number of tICS Components",box.lty=1, box.lwd=2, box.col="#000000")

dev.off()

postscript(paste0(nam,"diff.eps"),horizontal = FALSE,paper="special",height=24,width=20)
par(mfrow = c(6, 2))
par(cex = 1.5)
par(mar = c(2.5, 1.5, 1.75, 1.5), oma = c(2, 4, 0.5, 0.5))
par(tcl = -0.25)
par(mgp = c(2, 0.95, 0))

for(i in 1:p){
  cervplot = matrix(data=NA, nrow=n, ncol=3)
  tmp <- 0
  for(k in 1:3){
    tmp <- tmp + inv[i,k] * U[,k]
```

```
    cervplot[,k] = tmp - D[,i] + mean(D[,i])
  }

  cervplot.ts <- ts(as.matrix(cervplot),start=str)
  titnam <- paste("Age ",lab[i],sep="")

  ts.plot(cervplot.ts,gpars=list(xlab=NA,ylab=NA,axes=FALSE,ylim=c(-30,25),
                              col=c("#0000FF","#006400","#FF0000","#000000"),
                              lty=c(3,4,2,1),lwd=c(3,8,3,3)))
  abline(a=0,b=0,lwd=5,col="#000000")

  title(titnam,cex.main=2,font.main=1)
  axis(side=2,cex.axis = 1.75)
  axis(side=1,labels=ticsvec2,at=ticsvec2,cex.axis = 1.75)
  box()

}
mtext("Incidence", side = 2, outer = TRUE, cex = 4, line = 2.2,col = "#000000")
reset()

legend(x=xplotc,y=yplotc, legend = c(1:3),
      col=c("#0000FF","#006400","#FF0000"),lwd=12,
      lty=c(3,4,2),horiz = TRUE,
      cex = 3,title="Number of tICS Components",box.lty=1, box.lwd=2, box.col="#000000")

dev.off()

postscript(paste0(nam,"cluster.eps"),horizontal = FALSE,paper="special",height=10,width=25)
par(mfrow = c(1, 3))
par(cex = 1.5)
par(mar = c(4, 1.5, 2.1, 1.5), oma = c(4, 4, 0.5, 0.5))
```

```
  par(tcl = -0.25)
  par(mgp = c(2, 0.6, 0))
  for(k in 1:3){
    plotnam <- paste(nam,"comp",i,".eps",sep="")
    titnam<- bquote(paste("tICS Component ",.(k),", ",tau, "=",.(tau),sep=""))

    cervplot = matrix(data=NA, nrow=n, ncol=p)

    for(i in 1:p){
      cervplot[,i] = as.matrix(inv[i,k]*U[,k])
    }
    cervplot.ts <- ts(cervplot,start=str)
    ts.plot(cervplot.ts,gpars=list(col=col,axes=FALSE,type="l",lwd=6,xlab=NA,ylab=NA))
    title(titnam,cex.main=2.5)
    axis(side=2,cex.axis = 1.75)
    axis(side=1,labels=ticsvec2,at=ticsvec2,cex.axis = 1.75)
    box()
  }
  reset()
  legend(x="bottom",legend = c(lab), col=c(col),
         pch=16,ncol=5,bty="n", cex = 3.5)
  dev.off()

}
```

## **Cervical-Run.R**

```
########################################################################
#
# R-code that contains the generation of the figures of the paper
# "On Exploring Hidden Structures Behind Cervical Cancer Incidence"
#
# Author:
# email:
########################################################################

# Set the working directory to path where 'Cervical-Fun.R' and 'cervical2014.csv' are
setwd("...")

# The necessary functions for AMUSE and the plots
source("Cervical-Fun.R")

###########################
# Main paper, Figures 1-5
###########################

# Read the data into R workspace
Cerv <- read.csv("cervical2014.csv",sep=",",header=TRUE,row.names=1)
# 'cervical2014.csv' collected from NORDCAN

# Combine the age groups of older than 75 together
Cerv.Comb <- cbind(Cerv[,1:9], (Cerv[,10]+Cerv[,11]))

# Labels for the different age groups
lab1 <- c("0-34","35-39","40-44","45-49","50-54",
        "55-59","60-64","65-69","70-74","75+")
```

```
colnames(Cerv.Comb) <- lab1

# Set the colors for the curves
cols <- c("#FF1493","#8B008B","#00008B","#0000FF","#66CDAA",
        "#00FF00","#FFFF00","#FFA500","#FF0000","#838B8B")

# Convert the data into a time series object
Cerv.ts <- ts(as.matrix(Cerv.Comb),start=1953)

# Perform the AMUSE procedure with tau=1
Cerv.AMUSE <- NAMUSE(Cerv.ts,1)

AMUSE.D <- Cerv.AMUSE$Data
AMUSE.G <- Cerv.AMUSE$Gamma

# Reverse the sign of the first component (to make it match the mean curve)
AMUSE.Dmod <- AMUSE.D
AMUSE.Gmod <- AMUSE.G

AMUSE.Dmod[,1] <- -AMUSE.D[,1]
AMUSE.Gmod[1,] <- -AMUSE.G[1,]

# canplot <- function(D,U,UM,col,lab,ncom,tau,nam)
#    D       -       Original time series
#    U       -       AMUSE transformed data
#    UM      -       AMUSE transformation matrix
#    col     -       Vector containing the colors for the curves
#    lab     -       Vector containing the labels for the titles and legends
#    ncom    -       Number of tICS component plot figures generated
#    tau     -       Lag parameter of the AMUSE transformation (used in the titles of the figures)
```

```
#     nam          -              Name for the output figures

# Generates figures 1-5 (of the main paper) to your current working directory
canplot(Cerv.ts,AMUSE.Dmod,AMUSE.Gmod,cols,lab1,3,1,"cerv")

##########################
# Supplementary, Figure 1
##########################

# The first three components have the correct order (largest absolute eigenvalues),
# for we order the rest for the Appendix

ord1 <- order(abs(diag(acov(AMUSE.Dmod,1))),decreasing=TRUE)

AMUSE.D1 <- AMUSE.Dmod[,ord1]
AMUSE.G1 <- AMUSE.Gmod[ord1,]

canplot(Cerv.ts,AMUSE.D1,AMUSE.G1,cols,lab1,10,1,"sup1")

###############################
# Supplementary Figures 2,6,10
###############################

# Perform the AMUSE procedure with tau=2
Cerv.AMUSE2 <- NAMUSE(Cerv.ts,2)

AMUSE.D2 <- Cerv.AMUSE2$Data
AMUSE.G2 <- Cerv.AMUSE2$Gamma

ord2 <- order(abs(diag(acov(AMUSE.D2,2))),decreasing=TRUE)
```

```
AMUSE.D2ord <- AMUSE.D2[,ord2]
AMUSE.G2ord <- AMUSE.G2[ord2,]

canplot(Cerv.ts,AMUSE.D2ord,AMUSE.G2ord,cols,lab1,10,2,"sup2")

################################
# Supplementary, Figures 3,7,11
################################

# Perform the AMUSE procedure with tau=3
Cerv.AMUSE5 <- NAMUSE(Cerv.ts,3)

AMUSE.D5 <- Cerv.AMUSE5$Data
AMUSE.G5 <- Cerv.AMUSE5$Gamma

ord5 <- order(abs(diag(acov(AMUSE.D5,3))),decreasing=TRUE)

AMUSE.D5ord <- AMUSE.D5[,ord5]
AMUSE.G5ord <- AMUSE.G5[ord5,]

canplot(Cerv.ts,AMUSE.D5ord,AMUSE.G5ord,cols,lab1,10,3,"sup5")



################################
# Supplementary, Figures 4,8,12
################################

# Perform the AMUSE procedure with tau=5
Cerv.AMUSE3 <- NAMUSE(Cerv.ts,5)

AMUSE.D3 <- Cerv.AMUSE3$Data
```

```
AMUSE.G3 <- Cerv.AMUSE3$Gamma

ord3 <- order(abs(diag(acov(AMUSE.D3,5))),decreasing=TRUE)

AMUSE.D3ord <- AMUSE.D3[,ord3]
AMUSE.G3ord <- AMUSE.G3[ord3,]

canplot(Cerv.ts,AMUSE.D3ord,AMUSE.G3ord,cols,lab1,10,5,"sup3")

################################
# Supplementary, Figures 5,9,13
################################

# Perform the AMUSE procedure with tau=15
Cerv.AMUSE4 <- NAMUSE(Cerv.ts,15)

AMUSE.D4 <- Cerv.AMUSE4$Data
AMUSE.G4 <- Cerv.AMUSE4$Gamma

ord4 <- order(abs(diag(acov(AMUSE.D4,15))),decreasing=TRUE)

AMUSE.D4ord <- AMUSE.D3[,ord4]
AMUSE.G4ord <- AMUSE.G3[ord4,]

canplot(Cerv.ts,AMUSE.D4ord,AMUSE.G4ord,cols,lab1,10,15,"sup4")
```
